# Ribociclib Nanostructured Lipid Carrier Aimed for Breast Cancer: Formulation Optimization, Attenuating *In Vitro* Specification, and *In Vivo* Scrutinization

**DOI:** 10.1155/2022/6009309

**Published:** 2022-02-03

**Authors:** Ali Sartaj, Annu, Largee Biswas, Anita Kamra Verma, P. K. Sahoo, Sanjula Baboota, Javed Ali

**Affiliations:** ^1^Department of Pharmaceutics, School of Pharmaceutical Education and Research, Jamia Hamdard, New Delhi 110062, India; ^2^Nanobiotech Lab, Kirori Mal College, University of Delhi, Delhi 110007, India; ^3^Department of Pharmaceutics, Delhi Institute of Pharmaceutical Sciences and Research, New Delhi 110017, India

## Abstract

**Purpose:**

The current investigation is on the explicit development and evaluation of nanostructured lipidic carriers (NLCs) through the oral route to overcome the inherent lacuna of chemotherapeutic drug, in which Ribociclib (RBO) was used for breast cancer to diminish the bioavailability issue.

**Method:**

The RBO-NLCs were prepared using the solvent evaporation method and optimized method by the Box–Behnken design (BBD). Various assessment parameters characterized the optimized formulation and their *in vivo* study.

**Results:**

The prepared NLCs exhibited mean particle size of 114.23 ± 2.75 nm, mean polydispersity index of 0.649 ± 0.043, and high entrapment efficiency of 87.7 ± 1.79%. The structural analysis by TEM revealed the spherical size of NLCs and uniform drug distribution. An *in vitro* drug release study was established through the 0.1 N HCl pH 1.2, acetate buffer pH 4.5, and phosphate buffer pH 6.8 with % cumulative drug release of 86.71 ± 8.14, 85.82 ± 4.58, and 70.98 ± 5.69%, was found respectively, compared with the RBO suspension (RBO-SUS). *In vitro* intestinal gut permeation studies unveiled a 1.95-fold gain in gut permeation by RBO-NLCs compared with RBO-SUS. *In vitro* lipolysis suggests the drug availability at the absorption site. *In vitro* haemolysis study suggests the compatibility of NLCs to red blood cells compared to the suspension of the pure drug. The confocal study revealed the depth of penetration of the drug into the intestine by RBO-NLCs which was enhanced compared to RBO-SUS. A cell line study was done in MCF-7 and significantly reduced the IC_50_ value compared to the pure drug. The *in vivo* parameters suggested the enhanced bioavailability by 3.54 times of RBO-NLCs as compared to RBO-SUS.

**Conclusion:**

The *in vitro*, ex vivo, and *in vivo* results showed a prominent potential for bioavailability enhancement of RBO and effective breast cancer therapy.

## 1. Introduction

**Figure 1:**
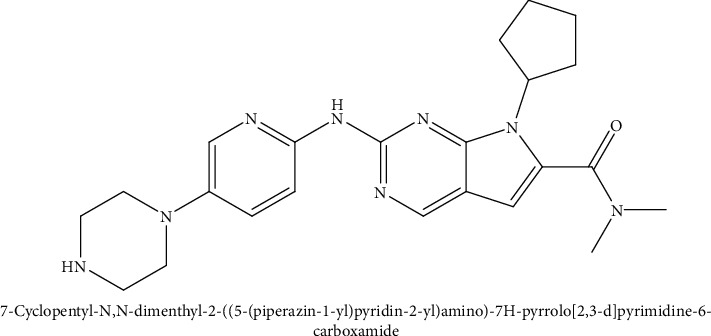
Chemical structure of Ribociclib with its IUPAC name.

The incidence of cancerous disease recently increased in recent years, impacting the physical, mental, and social life of humans. The incidence varies from developed countries (1 to 2%) to fewer development countries (almost 5%) yearly. The estimate also showed that more than 7 million people die from cancer disease and is predicted to be 10 to 15 million new cases added by 2020. Meanwhile, this disease is an unexceptional malignant breast cancer found in women, with more than 1 million fresh cases added yearly [1]. Breast cancer was ranked as number 1 due to the number in Indian women with an age rate as high 25.8 per 100,000 and a mortality rate of 2.7 per 100,000 women [2]. There are lots of chemotherapy drugs available for breast cancer; however, Ribociclib (RBO) is one of them that has been recently approved by the US FDA, and it is given orally of 200 mg as shown in Figure 1. The mechanism of action of RBO is to hamper the activity of cyclin-dependent kinase (CDK) at different types of cyclins like CDK4 and CDK6 of cell cycle pathways which indirectly inhibit the retinoblastoma (Rb) protein phosphorylation. The hampering of Rb again slows down the CDK-mediated G1-S phase movement. Because of this, the cell cycle is apprehended, inhibiting the cancer cell growth [3, 4]. The RBO is associated with various adverse effects, and the most common reaction, with more than 20% incidence, is neutropenia, nausea, fatigue, diarrhea, leukopenia, alopecia, vomiting, constipation, headache, and back pain. Apart from adverse effect, the drug is also associated with low solubility and permeability as it is a BCS class IV drug and undergoes immense metabolism by hepatic cells mediated via CYP3A4 in the human liver. Ribociclib is also the substrate for P-gp efflux, which leads to an increase in the dose to achieve the therapeutic level [5]. Thus, these major factors which decrease the degree of absorption of Ribociclib lead to its poor *in vivo* prospects. The clinical efficacy of Ribociclib is not satisfactory at the therapeutic level, which often results in an increase in dose frequency, or high dose causes adverse effects [6]. However, being a recently approved drug (RBO), not much work has been done so far in the nanocarrier system to overcome the related issue. A recent study investigated by Fei and Yoosefian on the preparation of RBO micelles to increase the aqueous solubility of hydrophobic drug carried out using dodecylphosphocholine surfactant [7]. The remaining challenge still to overcome is the bioavailability issue by inhibiting P-gp efflux and bypassing the extensive first-pass metabolism [8]. The lipidic nanocarriers are being developed in order to overcome the predicaments like improving solubility, minimization of P-gp efflux, protection of the drug from enzymatic (CYP3A4) degradation, providing metabolic stability (stability in GIT), enhancing permeability and retention in the target tissue, enhancing the bioavailability, and minimization of toxic drug effects [9]. The drug integrated into nanostructured lipidic carrier (NLCs) formulations with specific lipid (Solid and Liquid lipids) and excipients can regulate P-gp-mediated efflux movement and has the potential to change the pharmacokinetics of Ribociclib largely. In addition, lipids used in Ribociclib-loaded NLCs will promote its systemic blood circulation via the intestinal lymphatic system rather than being directly absorbed into the portal circulation and consequently prevent its significant first-pass metabolism [10]. Thus, our objective is to develop NLCs as a formulation which exhibits great potential for improving and normalizing the absorption of Ribociclib and contributing significantly in enhancing its therapeutic efficacy up to a satisfactory level.

## 2. Materials

RBO was obtained as gift samples from Alembic Pharmaceuticals Pvt. Ltd., Hyderabad, India. Various emulsifiers, oils, and lipids such as Labrafil M 1944 CS, Labrafil M 2125 CS, Maisine 351, Campritol 888 ATO, Precirol ATO 5, Gelot 64, Gelucire 44/14, Gelucire 44/01, and Solutol HS 15 were procured from Gattefosse India Pvt. Ltd. Capmul MCM, Capmul PG-12, Caprol PGE-860, were obtained from Abitec Corporation Cleveland, USA. Poloxamer 188, Cremophor EL and RH were obtained from BASF Mumbai, India. Tween 20 and Tween 80 were procured from Merck, Germany. Distilled deionized water used obtained from Milli-Q (Millipore, Bedford, MA, USA). The reagents used in all methods were of analytical grade available in the lab facility.

## 3. Methods

### 3.1. Assessment of Excipients

#### 3.1.1. Evaluation and Selection of Liquid Lipids

The selection process for liquid lipid encompassed taking 2 ml of liquid lipid in a glass vial to which a surplus quantity of drug was added to evaluate the maximum solubility. The glass vials containing lipid and drug were tightly stoppered and maintained on a mechanical shaker for 72 h at 25.0 ± 0.5°C. This was further centrifuged at 5000 rcf, and the supernatant was dissolved in methanol before quantitatively analyzing using a UV-spectroscopy at a wavelength of 282 nm [11].

#### 3.1.2. Evaluation and Selection of Solid Lipids

Similarly, for solid lipid selection, the known quantity, i.e., 100 mg of the drug, was taken in a glass vial to which solid lipid was added up in surplus amount slowly until the solid lipid solubilizes the drug. The temperature was maintained at higher than the actual melting point (5°C above the melting point) of each solid lipid. The amount wherein an unclouded solution has been seen was taken as the endpoint [11].

#### 3.1.3. Compatibility of Solid and Liquid Lipids

To determine the solid and liquid lipid ratio and establish their compatibility, different ratios of 6 : 4, 7 : 3, 8 : 2, and 9 : 1 were undertaken and evaluated for phase separation. The binary mixture was retained at an elevated temperature compared to the melting point (5°C above the melting point) of each solid lipid for the study. The mixture demonstrating no evidence of phase separation for 24 hours was selected [12].

#### 3.1.4. Thermal Inspection of a Binary Mixture by DSC

The lipids of either category with inflated solubilization of RBO were taken. The different ratios of binary mixture with a surplus amount of liquid lipid were prepared. The ratio of solid : liquid lipid in 6 : 4, 7 : 3, 8 : 2, 9 : 1, and 1 : 0 was prepared. The binary mixture was held onto a water bath at an elevated temperature compared to the melting point of solid lipids, i.e., 5°C above the melting point with continuous agitation. The final sample holds on to room temperature for 24 hours. Afterward, all binary mixture samples were analyzed using DSC (Perkin Elmer Pyres, USA) thermogram. The change in any thermal incident of solid lipid alone was considered as a control. The instrument was set at a 10°C heating rate per minute, and the temperature range was selected from 25°C to 100°C. The crystallinity index (CI) was determined for each ratio of the binary mixture sample [13].

#### 3.1.5. Screening of Surfactant

The optimized ratio of solid : liquid lipid (100 mg) was dissolved in 3 ml of methylene chloride. The methylene chloride was separated later by moderate heating. To the above organic mixture, 10 ml of respective surfactant solutions of 5% *w*/*v* concentration was added and allowed to stir for 30 min. 1 ml of the sample was taken and further diluted to 10 times, and then, % transmittance was taken at 510 nm in UV spectroscopy [13].

### 3.2. Preparation and Optimization of NLCs

For the development of NLCs, solvent evaporation followed by the probe sonication method was employed. The drug was dissolved in the lipidic binary mixture (solid : liquid lipid mixture) to which the surfactant was added dropwise, maintained at the same temperature. This was continuously stirred for 30 min at 200 rcf followed by the probe sonication method, which was used for size reduction [11]. To obtain the optimized formulation, the QbD technique was applied wherein Box-Behnken Design (BBD) was used considering the two variables as dependent and independent. For optimization techniques, Design-Expert software was used. Three factorial designs were applied with 3 levels, which lead to a total of 17 experiments. These trials were also giving a needed quadratic equation to yield the optimized formula as undermentioned.

(1) $$\begin{array}{c} Y=c_{0}+c_{1}X_{1}+c_{2}X_{2}+c_{3}X_{3}+c_{12}X_{1}X_{2}+c_{13}X_{1}X_{3}+c_{23}X_{2}X_{3}+c_{11}X_{1}^{2}+c_{22}X_{2}^{2}+c_{33}X_{3}^{2}. \end{array}$$

In the above equation, *Y* represents the computed response association of factor level; *c*_0_ is the constant; *c*_1_, *c*_2_, and *c*_3_ are linear coefficients; *c*_12_, *c*_13_, and *c*_23_ are interaction coefficients in the midst of 3 factors, whereas *c*_11_, *c*_22_, and *c*_33_ represent observed experimental results of quadratic coefficients; and independent variable values are represented by *A*_1_, *A*_2_, and *A*_3_ [14]. The independent variable for the said process for BBD was a binary mixture (1-3%), surfactant % (1-3%), and sonication time (1-5 min). The dependent variables include the particle size as well as PDI and entrapment efficiency.

### 3.3. Characterization Parameters of Optimized RBO-NLCs

#### 3.3.1. Particle Size and Polydispersity Index (PDI)

Zetasizer Nano ZS (Malvern, UK) instrument used to determine the particle size distribution and PDI, which is based on the dynamic light scattering principle. Zetasizer calculates the variation in the intensity of light transfer attributable to the Brownian motion of the lipid nanoparticulate carrier system [15].

#### 3.3.2. Surface Charge Determination

The surface charge of optimizing RBO-NLCs was measured in terms of zeta potential by the Zetasizer (Malvern, UK) instrument. The instrument was calibrated by the conductivity to 50 mS/cm with 0.9% *w*/*v* solution of sodium chloride against the purified water. The zeta potential was measured after dilution of the sample with distilled water (approx. 50 *μ*l of sample and diluted to make up to 10 ml volume with distilled water). The diluted sample was used to determine the zeta potential at 25 ± 2°C [16].

#### 3.3.3. Drug Entrapment (EE) and Drug Loading (DL)

The prepared RBO-NLCs were centrifuged (Sigma, Germany) at a speed of 10,000 rpm (5590 × *g*) for half an hour. The supernatant was separated, and the pellets present at the bottom were collected. The pellet was further diluted with 10 ml methanol. After dilution, mixed well and then analyzed by UV-spectroscopy method [17]. Therefore, the amount of encapsulated drug is analyzed by the amount of drug present in pellets. The following formulas were used to determine each of the parameters:

(2) $$\begin{array}{c} \left(\%\right)\text{EE}=\frac{W_{\text{total drug}}–W_{\text{free drug}}}{W_{\text{total drug}}}\times 100\%,\\ \left(\%\right)\text{DL}=\frac{W_{\text{total drug}}–W_{\text{free drug}}}{W_{\text{total lipid}}}\times 100\%, \end{array}$$

where *W*_total drug_ was the total amount of drug, *W*_free drug_ was the amount of unencapsulated drug, and *W*_total lipid_ was the weight of the lipids used for NLCs preparation.

### 3.4. Freeze-Drying of NLCs Formulation

Mannitol was used as a cryoprotectant and added to the optimized RBO-NLCs. The obtained dispersion solution after adding mannitol was kept at a frozen temperature (-20°C) overnight. Then, a sample of frozen dispersion was lyophilized at -20°C for 12 h in a freeze dryer (Make Heto Drywinner, Denmark). The free-flowing powder was obtained after freeze-drying used for different characterization parameters during studies [18].

### 3.5. Structural Analysis by TEM

TEM (Fei Company, Netherlands) was used for the ascertainment of shape and size of optimized NLCs formulation. Before analyzing, the sample of RBO-NLCs was prepared after diluting it 10 times with distilled water onto a copper grid mesh of 400 (coated with carbon film). Then, 1% of phosphotungstic acid was added to the copper grid as negative staining. The sample above the copper grid was dried in the air. Then, the dried sample was examined in TEM. The setup of elevated magnification coupled with diffraction modes inside the instrument was used to examine the shape and size of the optimized NLCs using brightfield imaging [19].

### 3.6. Powder X-Ray Diffraction (PXRD) Pattern Study

For PXRD analysis, a Rigaku Ultima IV (Tokyo, Japan) instrument was used with a degree divergence slit, degree scatter slit, and receiving slit, set at 40 kV to determine the crystallinity form of pure drug and RBO-NLCs to confirm whether the drug was entrapped into the NLCs formulation or not. Scans were performed from 10 to 80° 2 thetas at 8°/min at 25° room temperature with 65-75% humidity.

### 3.7. Structural Analysis by FT-IR Spectroscopy

The FT-IR (Bruker, Tensor 37, Germany) spectra of drug alone and NLCs formulation were recorded. The powder sample of each sample was mixed with Potassium Bromide (KBr) in a ratio of 1 : 100. The final sample was pressed into pellets using a hydraulic pressure machine.

### 3.8. Drug Release Study

#### 3.8.1. Activation of Dialysis Membrane

Activation of the dialysis membrane is required before *in vitro* study to remove glycerine and sulfur compounds and to open pores. The continuous washing for 3-4 h in running water removed the glycerine. The solution of 0.3% *w*/*v* sodium sulfide was used to wash at 80°C for 1 min to remove the sulfur compounds. The third step was to wash with hot water (60°C) for 2 min to remove the extra sodium sulfide solution and sulfur compound. The dialysis bag was further treated with sulfuric acid (0.2% *v*/*v*) for acidification and finally washed with hot water to remove extra sulfuric acid [20].

#### 3.8.2. *In Vitro* Drug Release Study

The drug release from RBO-NLCs was performed in various buffers like 0.1 N HCl buffer pH 1.2, acetate buffer (AB) pH 4.5, and phosphate buffer saline (PBS) pH 6.8 by dialysis technique. The 3 ml of RBO-SUS and RBO-NLCs freshly prepared formulation was taken. Then, each sample of 3 ml was placed in activated dialysis bags (molecular weight cut-off 12000 g/mole), and both ends were tied to form a sac-like structure. The dialysis bag is suspended in dissolution media of 100 ml in the above-mentioned various buffers at 37 ± 0.5°C. The magnetic stirring rate was maintained at 100 rpm. The aliquot sample of 2 ml was withdrawn at regular intervals of time from dissolution media (5 min, 1, 2, 4, 6, 8, 12, 24, 32, 48, and 72 h). The withdrawn samples were diluted with respective dissolution media. The diluted sample was used to measure the quantity of drug-using UV-spectroscopy. The drug release was calculated as per the formula given. The dissolution studies were performed in triplicate. The dissolution profile of each formulation was compared in terms of % dissolution efficiency (%DE) and similarity factor (f2) [21].

(3) $$\begin{array}{c} \%\text{Drug release}=\frac{\text{Conc}.\left(\mu \text{g}/\text{ml}\right)\times \text{Dilution factor}\times \text{Volume of release medium }\left(\text{ml}\right)}{\text{Initial dose }\left(\mu \text{g}\right)}\times 100. \end{array}$$

#### 3.8.3. Drug Release Kinetic Models

The release profile of the drug encapsulated into NLCs over time was used to fit into various release kinetic models like the first order, Higuchi model, Hixson-Crowell, and Korsmeyer-Peppas, as mentioned in Table 1 with their respective equations. Each equation is used to calculate the correlation coefficient (*R*^2^). The *R*^2^ value close to 1 was selected as the best-fit kinetic model for drug release. The *n* value present in the equation indicates their mechanism of drug release, which varies from 0.43 to 0.85. If *n* is 0.43 or less, it follows the Fickian diffusion. If *n* value 0.43 < *n* > 0.85, then it follows the non-Fickian mechanism. If the *n* value is 0.85, then it follows the zero-order mechanism of drug release [22].

### 3.9. *In Vitro* Lipolysis

This study was performed to establish the solubilization potential of NLCs when given orally and the result interpreted for *in vivo* fate. The composition of different media used for *in vitro* lipolysis like digestive buffer (17.75 ml) made of 50 mM tris-maleate, 5 mM calcium chloride, 150 mM sodium chloride, and 39.75 mM sodium hydroxide. The pH of the digestive buffer was adjusted to 6.8 by 1 M sodium hydroxide. To the above buffer, 5 mM taurocholic acid and 1.25 mM L-alpha phosphatidylcholine were added. The resulting solution mimics the fasted state of GIT. The buffer was stirred and maintained at a temperature of 37°C, and the pH 6.8 was adjusted by 1 M sodium hydroxide. The NLCs formulation of 5 mg per ml drug concentration was taken and mixed to the digestive buffer and stirred for 15 min. Another media of pancreatin extract (1.75 ml) was added to the above digestive buffer containing the NLCs formulation made of porcine pancreatin of 200 mg per ml concentration in the digestive buffer. After adding the pancreatin extract, the media were well stirred for 15 min. The enzymatic digestion of RBO-NLCs was initiated, and accordingly, pH was adjusted by 0.15 M sodium hydroxide to pH 6.8. The whole setup was recorded for 30 min. The volume required to maintain the pH 6.8 which corresponded to the release of 2 free fatty acids was calculated. The final mixture was centrifuge at 5000 rpm for 15 min after the end of the experiment, with the supernatant comprising the aqueous layer containing bile salts, monoglycerides, and fatty acids. The sediment comprises lipid-containing di- and triglycerides and undissolved fatty acid. The drug content was measured in each separated layer after centrifugation [23].

### 3.10. *In Vitro* Haemolysis

Haemolysis studies were performed to see the compatibility of RBCs with NLCs formulation compared to their pure drug suspension. Freshly human blood was collected in an EDTA tube and centrifuge for 5000 rpm for 10 min and supernatant was decanted. The remaining solid content was washed 3 times with phosphate buffer (pH 7.4). After washing with phosphate buffer, centrifuge at 5000 rpm for 10 min and the solid content was taken after decantation. An appropriate amount of NLCs formulation of 20 *μ*L and diluted RBCs with phosphate buffer of 180 *μ*L were taken in 96 well plates. Similarly, 20 *μ*L of NLCs placebo, pure drug suspension, 1% triton X100 (negative control) and phosphate buffer (positive control) were taken at respective 96 well plates. The optical density was measured by the Elisa reader at 570 nm after one hour from the sample added to diluted RBCs (RBCs in phosphate buffer). Furthermore, percentage haemolysis was calculated by the given formula [24]:

(4) $$\begin{array}{c} \text{Haemolytic activity }\left(\%\right)=\left\{\frac{\text{As}–\text{Ab}}{\text{Ac}}\right\}100, \end{array}$$

where As, Ab, and Ac are the optical density of the sample, placebo, and positive control, respectively.

### 3.11. *In Vitro* Gut Permeation Study

The small intestine of Wistar rat (Protocol No. 1587. from IAEC, Jamia Hamdard) was used for *in vitro* gut permeation study, which was fasted overnight. All animal experiments comply with the ARRIVE guidelines. The CO_2_ inhalation is used to sacrifice the rat. The small intestine was isolated into 4-5 cm length. The isolated intestine was washed with Tyrode buffer pH 7.4 to clean the content of the intestine. The intestinal part of one end is tied with thread, and 3 ml of RBO-NLCs is filled using a syringe from the second end and tied. The sac formed after tying from both ends is immersed into a glass beaker maintained at 37°C, which contains oxygenated Tyrode buffer pH 7.4 (100 ml). The aliquot of 2 ml was withdrawn at regular intervals of time like 5 min, 10 min, 15 min, 30 min, 1 h, 1.5 h, and 2 h and replaced with similar preheated dissolution media.

Similarly, the experiment was done for RBO-SUS. The aliquot taken from each sample at regular intervals of time was analyzed for drug content by HPLC [25]. The flux and apparent permeability coefficient were calculated using the following formula:

(5) $$\begin{array}{c} \text{Flux of drug}=\frac{\text{Conc}.\left(\mu \text{g}/\text{ml}\right)\times \text{Dilution factor}\times \text{Volume of release medium }\left(\text{ml}\right)}{\text{Permeation area }\left(\text{c}{\text{m}}^{2}\right)}\times 100,\\ \text{Apparent permeability coefficient},P\left(\text{cm }\min^{-1}\right)=\frac{J}{C_{o}}, \end{array}$$

where *C*_*o*_ is the initial quantity of the drug present in the donor compartment.

### 3.12. Depth Permeation Study by Confocal Microscopy

The experiment assessed the depth of penetration of RBO-NLCs compared to drug suspension in small intestinal gut cells. The NLCs formulation and drug suspension were added with Rhodamine B dye (0.03%) during the preparation of each formulation. The experiment kept for 2 h as mentioned in section 3.11. Then intestinal gut samples were separated and cleaned with Tyrode solution, pH 7.4. The cleaned intestinal gut was subjected cut off into thin sections and fixed over the slide for further analysis. The thin section was examined by confocal microscopy (LEICA TCS SPEII Leica Microsystem Ltd., Germany) with LAS AF software. Fluorescence indicator from cells of the intestine was examined at various depths of the intestinal membrane. The magnitude and depth of permeation of fluorescence in intestinal membrane cells were calculated through the *z*-axis and compared the result [26].

### 3.13. Cell Line Study

#### 3.13.1. Cell Line and Cell Culture

The MCF-7 BC cell lines purchased from National Centre for Cell Science, Pune, India (Job No. 408/20220.21), were cultured in DMEM supplemented with composition 10% (*v*/*v*) fetal bovine serum (FBS) and 1% (0.01 g/mL) penicillin-streptomycin solution maintained at 37°C temperature in a humidified incubator of <5% CO_2_ and 95% humidity.

#### 3.13.2. Determination of Cell Cytotoxicity by MTT Assay

The cytotoxicity of the RBO at concentrations of 12.5, 25, 50, and 100 *μ*M was evaluated as per the previously published protocol, and percent cytotoxicity was calculated in the MCF-7 cell line. Briefly, 5 × 10^3^ cells per well were seeded in a 96-well microtiter plate containing 10% FBS and kept for incubation for 24 hrs maintained at 37°C with 5% CO_2_. The next day, the cells were treated with samples at mentioned concentrations for 24 hrs and 48 hrs. The 20 *μ*l of MTT solution made in PBS pH 7.4 of 5 mg/ml concentration was added to each well and remained for 4 hrs at 37°C which allowed the formation of formazan crystals. The crystals were again dissolved in DMSO by adding 150 *μ*l to each well. Finally, the content of the plate was well mixed on mechanical plate mixer and then optical density (OD) was measured in an ELISA reader (Synergy HT, BioTek, USA) at 570 nm. The colour developed in each well is directly related to the viable present in respective well. The 50% inhibition concentration (IC_50_) was determined after 24 hrs and 48 hrs, and percentage cytotoxicity was calculated as per the given formula. All experiments were done in triplicates [27].

(6) $$\begin{array}{c} \%\text{cytotoxicity}=\frac{{\left[\text{C}\right]}_{\text{control}}-{\left[\text{T}\right]}_{\text{test}}}{{\left[\text{C}\right]}_{\text{control}}}\times 100, \end{array}$$

where [*T*]_*test*_ is the Absorbance of test sample and [C]_control_ is the absorbance of the control sample.

The concentration value of RBO needed to inhibit the cell growth by 50% so that IC50 values were calculated using a dose-response curve.

### 3.14. *In Vivo* Pharmacokinetic Study

All experiments on the animal were conducted as per the guidelines of IAEC, Jamia Hamdard, New Delhi, India (Protocol no. 1587). The animals were kept under normal laboratory supply, i.e., temperature of 25 ± 2°C and relative humidity of 55 ± 5% RH with normal laboratory diet supply. For pharmacokinetic study, female Wistar rats were assigned randomly into two groups (*n* = 3). Among two groups, group A received RBO suspension (approx. 20 mg/kg of body weight) and group B received RBO-NLCs (approx. 20 mg/kg of body weight) calculated from human to animal dose conversion factor [28]. RBO being a water insoluble compound, the suspension of RBO was made with 0.25% *w*/*v* Na-CMC (suspending agent) with a constant stirring for 10 min so that the final concentration of suspension was 2.5 mg/ml of RBO. The similar method of suspension preparation has been reported by Singh and associates in which Na-CMC was added to water at temperature of 40 to 50°C under continuous stirring of 600 rpm, and the same has been followed for the current study [26].

Blood sample were collected from venous blood drawn from the lateral tail vein in EDTA-coated tubes in predetermined interval of times (0.5, 1, 2, 4, 8, 12, 24, and 48 h) under mild CO_2_ inhalation anaesthesia. The blood samples were centrifuged for 0.5 h at 5000 rpm to separate plasma from blood. The plasma samples were stored at -80°C until the further analysis by HPLC. The plasma samples were thawed out before analysis. For deproteinization, the plasma samples were mixed with acetonitrile (equal quantity of plasma so that 50% dilution). Then, the samples were centrifuged for 0.25 h at 5000 rpm, and supernatants were collected for quantification of RBO by HPLC [26].

### 3.15. Stability Study

The optimized RBO loaded into NLCs was stored at room temperature with set down conditions of 25 ± 2°C/60 ± 5% relative humidity as per Q1A(R2), ICH guideline. The change in any physical parameters like particle size and PDI and chemical parameters like entrapment efficiency monitored during the storage period of 6 months [29]

## 4. Results and Discussions

### 4.1. Assessment of Excipients

#### 4.1.1. Selection of Lipids

**Figure 2:**
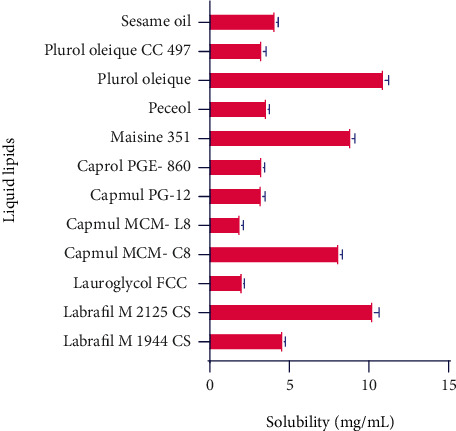
Solubility of RBO in liquid lipids.

**Figure 3:**
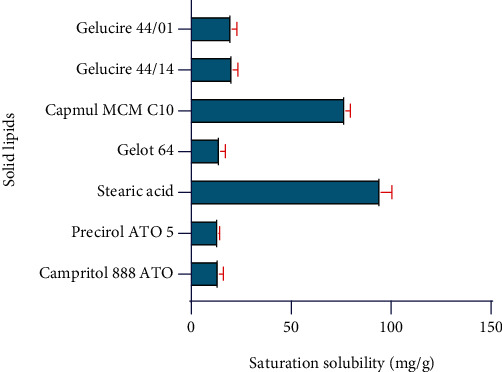
Solubility of RBO in solid lipids.

The selection of different required ingredients like lipids for the fabrication of NLCs was performed based on the maximum solubility of the drug in each ingredient. The solubility of RBO performed in several lipids and results are elaborated in Table 2 and Figures 2 and 3.

Based on solubility data, Labrafil M2125 CS (HLB 4), Capmul MCM C8 (HLB 5-6), Maisine 3S1 (HLB 4), and Plurol oleique (HLB 6) were showing high solubility of RBO as it was anticipated that the structure containing carboxylic acid and succinate group favors the solubility of RBO in each liquid lipid. In addition, longer triglycerides have shown more solubility than the medium-chain due to more entrapment of drugs. Similarly, Stearic acid (HLB 17) and Capmul MCM C10 (HLB 5) have shown high solubility of RBO due to similar reasons. The solubility parameters of each lipid direct reflect the encapsulation efficiency. Thus, the high solubility associated with the drug in lipids is also attributed to the high encapsulation efficiency of the NLCs formulation [29]. Bang and coworkers investigated NLCs of anticancer drug paclitaxel, and similar methods were applied for the selection of solid and liquid lipid for the NLCs preparation [30, 31].

#### 4.1.2. Compatibility Study of Lipids

The results of compatibility studies are given in Table 3. Labrafil M 2125 CS, Capmul MCM- C8, Maisine 351, Plurol oleique were selected as liquid lipids, and Stearic acid and Capmul MCM C10 were selected as solid lipids for further compatibility/miscibility study among lipids. Negi and coworkers develop a protocol for solid and liquid lipid-based compatibility on miscibility/affinity and the physical compatibility of lipids. So based on these parameters, Capmul MCM C8 and Compritol were selected [31].

#### 4.1.3. Thermal Inspection of a Binary Mixture by DSC

**Figure 4:**
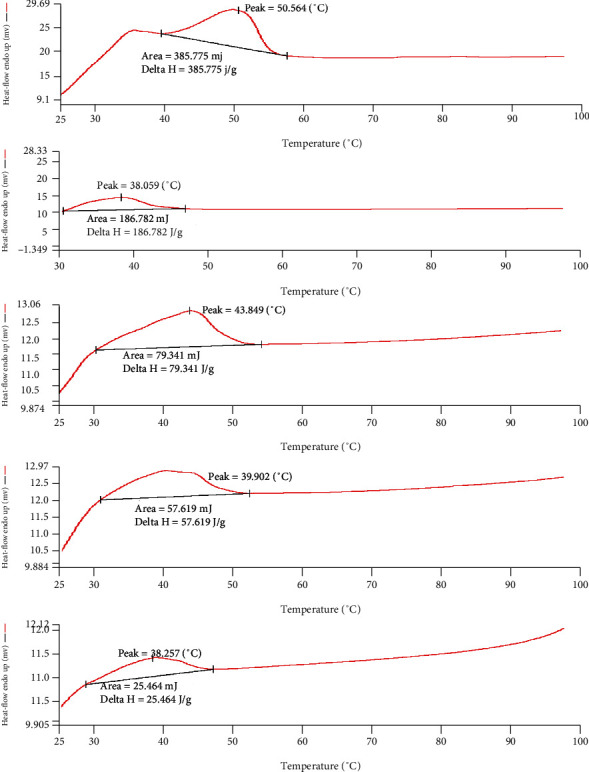
Compatibility study of liquid lipids and solid lipids.

**Figure 5:**
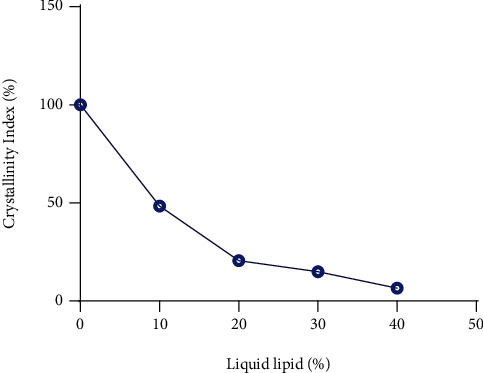
Determination of compatibility of lipids through Crystallinity Index (%).

In a ratio of the binary mixture, as the % of liquid lipid increases by 10%, the enthalpy for the melting point decreases in respect of the solid lipid (100%) as shown in Figure 4. However, the crystallinity index (CI) has less variability in the ratio of 8 : 2 to 6 : 4 compared to 9 : 1. Therefore, the final ratio was selected based upon CI, i.e., 6 : 4, as shown in Figure 5 [32]. Iqbal and associates reported similar parameters for the selection of the best ratio of solid and liquid lipids for a suitable binary mixture. As the % of the liquid lipid (Sefsol 218) increases, the enthalpy of the binary mixture decreases with respect to the solid lipid (Geleol), and it was accompanied by a decrease in crystallinity index from 100.0 to 30.3% [13].

#### 4.1.4. Selection of Surfactant

The surfactants, namely, Cremophor RH 40, Cremophor EL, Tween 20, Tween 80, Poloxamer 188, and Solutol HS15, were used to evaluate for its emulsification property. The result of the screening is illustrated in Table 4. The higher emulsification property reflects to reduce the particle size of binary mixture lipids and increase the physical stability of lipid nanoparticles, sequentially preventing the aggregation of nanoparticles which gives higher transmittance [33]. Alam and coworkers developed isradipine NLCs. They have reported in their research about the surfactant selection criteria based on % of transmittance, which was examined by UV spectroscopy method at 510 nm [12].

### 4.2. Preparation and Optimization of NLCs

With regard to approaches to Pharmaceutical Development, the product should be designed to meet needs and the intended product performance. A researcher might select either an experimental approach, an organized approach, or both for product development. A more organized approach for product development also refers to the quality by design approach, which includes adding prior knowledge and their results using the design of experiments (DOE). Such an organized approach helps to get the final desired product with the desired quality. It also helps to understand all the regulations. With respect to the quality of the product, which is generally related to safety and efficacy, setup parameters were justified, which refers to the quality target product profile (QTPP) which gives the outlook summary of drug product characterization, which can be predetermined. The dependent variables like particle size, entrapment efficiency, and PDI are considered critical quality attributes (CQAs). For effective delivery at the targeted site, the particle size should be small; more entrapment of the drug leads to therapeutic efficacy even at a low dose and low PDI (≤0.7) [34] which is considered as a uniformly distributed formulation. These parameters of QTPP and CQAs are summarized in Tables 5 and 6, respectively [35, 36].

#### 4.2.1. Risk Assessment

**Figure 6:**
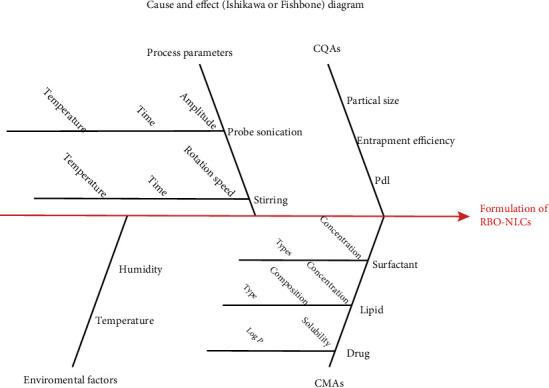
Ishikawa diagram to understand the relation of various factors.

The profound parameters for further experiment for optimization of formulation using Box-Behnken design from preliminary assessed data were binary mixture concentration (1-3%), surfactant concentration (1-3%), and sonication time (1-5 min). The 100% equipped response surface fraction is present in the design space to signify its accuracy all over the region.  Figure 6 demonstrated the relation among all factors for NLCs preparation prepared by software Minitab 19, Philadelphia, USA. Similarly, Kovacs and associates have reported for the salicylic acid-loaded NLCs in which they have investigated the risk assessment, which referred to both qualitative and quantitative estimates of the risk. An Ishikawa diagram was drawn to find out the key material and process attributes which affects the NLCs formulation preparation [37].

#### 4.2.2. Box-Behnken Design (BBD)

The Box-Behnken design (BBD) response surface method tested the points which are within a predetermined range unlike the central composite design (CCRD) to get the total possible runs based on the optimized NLCs formulation data, which was prepared by probe sonication technique; 17 runs were obtained using the mentioned variables in Table 7.  Table 8 gives the result derived from the experimental runs. The optimized formula obtained was a binary mixture, surfactant, and sonication time which were found as 1% *w*/*v*, 3% *w*/*v*, and 3 min, respectively. The results of the dependent variable for the optimized formula were particle size (114.23 ± 2.75 nm), entrapment efficiency (87.7 ± 1.79%*w*/*w*), and PDI (0.649 ± 0.043).

The quadratic equations obtained for different dependent variables and *p* value were found significant, and lack of fit has been found to be insignificant, as mentioned below in Table 9.

Particle Size (B1) = 219.44, *X* 148.23, *Y* -117.26, *Z* 9.16, *XY* -70.29, *XZ* 50.69, *YZ* -27.84, *X*^2^ 57.97, *Y*^2^ 37.42, *Z*^2^ 59.12

PDI (B2) = 0.5432, *X* 0.0363, *Y* 0.0216, *Z* 0.0081, *XY* -0.0455, *XZ* -0.0515, *YZ* 0.0198, *X*^2^ 0.0738, *Y*^2^ -0.0115, *Z*^2^ -0.0185

Entrapment efficiency (B3) = 89.12, *X* 4.49, *Y* 0.3900, *Z* -0.2888, *XY* -0.0450, *XZ* -0.0775, *YZ* 1.11, *X*^2^ 2.16, *Y*^2^ -0.2213, *Z*^2^ 0.0513

**Figure 7:**
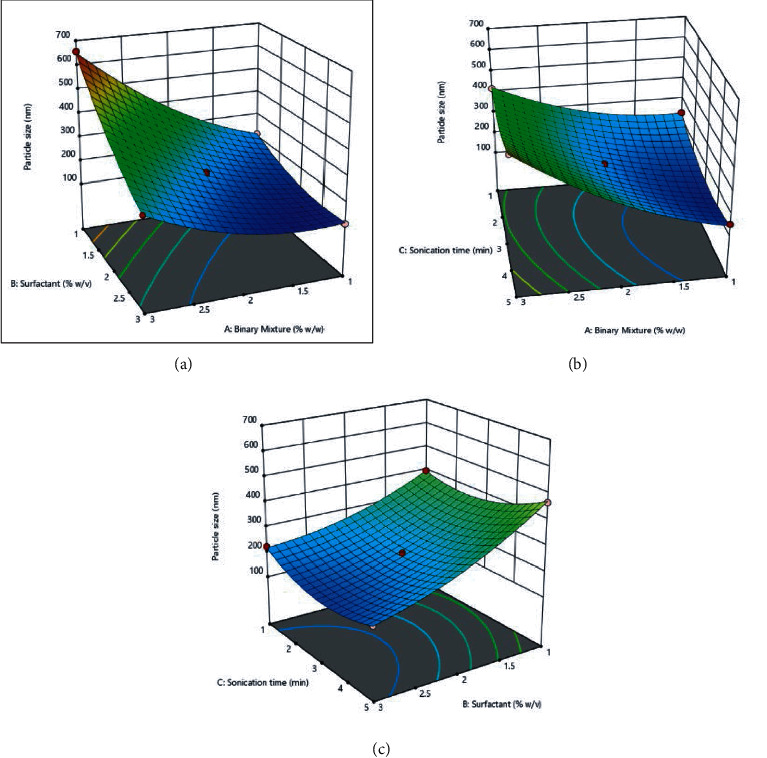
Effect of independent variables: (a) binary mixture and surfactant; (b) binary mixture and sonication; (c) surfactant and sonication on particle size.

**Figure 8:**
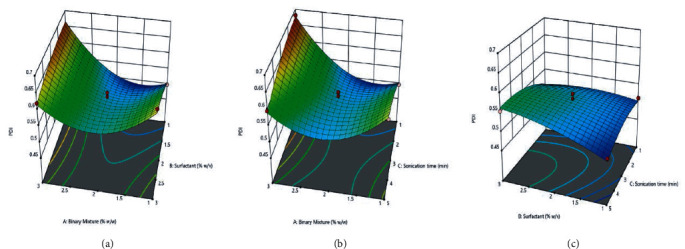
Effect of independent variables: (d) binary mixture and surfactant; (e) binary mixture and sonication; (f) surfactant and sonication on PDI.

**Figure 9:**
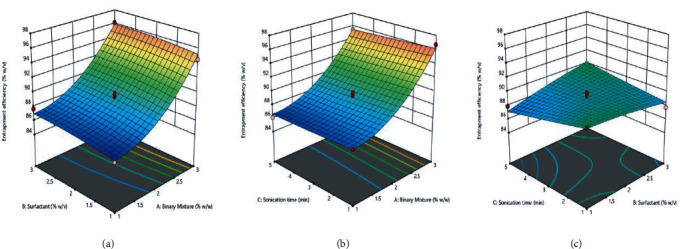
Effect of independent variables: (g) binary mixture and surfactant; (h) binary mixture and sonication; (i) surfactant and sonication on entrapment efficiency.

In all the equations, *A* represents the binary mixture %, *B* is the surfactant %, and *C* depicts the sonication time (in min). The effect between each category of variables was depicted using 3-dimensional graphs in response surface analysis, as shown in (Figures 7, 8, and 9).

#### 4.2.3. Impact of Independent Variables on Responses

*(1) Impact of the Binary Mixture on Dependent Variables*. As the binary mixture concentration increases, the particle size also increases because of agglomeration of particles due to lack of emulsifying agent (surfactant) as more emulsifying agent is required if lipid concentration increases aqueous phase, and lack of it is attributed to an increase in particle size.

Entrapment efficiency was directly affected binary mixture quantity. This indicated that an increase in the lipid concentration leads to an increase in the amount of solid lipid, which can be, accommodated more drugs inside the NLCs formulation in the solid lipid [38]. Similarly, Subedi and associates investigated the Doxorubicin loaded into the lipidic carrier, which stated the factor affecting the quantity of lipid on particle size as well as encapsulation efficiency. So, the increment of the lipid content increases the encapsulation efficiency and particle size [39].

*(2) Impact of Surfactant on Dependent Variables*. The lipid nanoparticle particle size decreases as the concentration of surfactant increases above the critical micelle concentration due to the decrease in interfacial tension. This critical concentration of surfactant also forms a network on the surface of lipid nanoparticles which prevent its agglomeration of the small particle from forming a bigger particle size.

However, the increase in the concentration of surfactant on entrapment efficiency was found very minimal [40]. The results are in agreement with the research reported by Shtay and associates. They have studied the factorial design, which applied to variables that affecting their preparation for food application using lipid nanocarrier. They have reported the increase in concentration decrease the particle size explained by a decrease in interfacial tension [40].

*(3) Impact of Sonication Time on Dependent Variables*. The mechanism of probe sonication is based on the principle that high energy waves ultrasonically emitted cause the breakage of lipid nanoparticles into small nanoparticles. Thus, upon an increase in sonication time, the lipid nanoparticle size decreases and forms monodisperse globules because of the shear force generated by cavitation.

However, the impact of sonication on entrapment efficiency was observed to be very minimal with aggravation in the sonication time [41]. Ahad and coworkers formulate the lipidic nanocarrier using an experimental design technique for valsartan. They reported an inverse relationship between the sonication time and vesicle size [42].

#### 4.2.4. Validation of Design of Experimental

The goal of the current study is to achieve minimum particle size with maximum entrapment efficiency. Design-Expert software, version 12.0.3.0 (Minneapolis, USA), was applied for the predicted value of response given in Table 10. Based on independent variable values, RBO-NLCs prepared and compared their data with a predicted response. The value obtained from the experiment was almost similar, which confirms the validity of the optimized process for preparing NLCs except for the particle size, which might be due to the wide variety of particle data obtained from a number of runs which give a broad mean value.

### 4.3. Characterization of Optimized RBO-NLCs

#### 4.3.1. Particle Size, PDI, and Surface Charge

The nanoparticle size (Z-Average) and PDI were obtained at 114.23 ± 2.75 nm and 0.649 ± 0.043, respectively. The zeta potential of RBO-NLCs was obtained at 2.61 ± 0.54 mV. Smaller particle size suggests good miscibility and more systemic circular time. The low PDI value states the uniformity of nanoparticle distribution. Zeta potential indicates the stability of the nanoformulation. Varshosaz and coworkers investigated the NLCs preparation in which the particle size found was smaller (103 to 127 nm) because their good miscibility of the excipient and zeta potential were positive values suggesting longer residence time and less uptake by the reticuloendothelial system in the blood circulation system [43].

#### 4.3.2. Drug Entrapment (EE) and Drug Loading (DL)

The % drug entrapment efficiency of NLCs was calculated to be 87.7 ± 1.79%*w*/*w*. The lipophilic drug has the intrinsic property to get more solubilization in lipids, unlike the hydrophilic drug. So, this lipophilic property entraps them more inside the lipids.

The % DL was found to be 8.77 ± 0.18%*w*/*w*. Elmowafy and coworkers investigated drug-loaded NLCs to overcome the oral delivery drawback. They have found the % encapsulation efficiency varies between 76 ± 12.4% and 96.6 ± 7.1%. The high % of liquid lipid (Capryol® PGMC) causes massive crystal order defects, which lead to more space to entrap the drug molecules and improve the % EE [44].

#### 4.3.3. Structural Analysis by TEM

**Figure 10:**
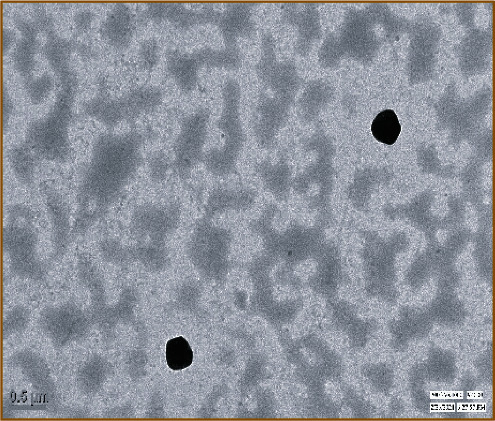
TEM image of optimized RBO-NLCs.

The TEM image shown in Figure 10 demonstrated that the RBO-NLCs particles were uniformly distributed with no sign of any aggregation of particles, and the shape of each nanoparticle was spherical. The result indicated that the NLCs particle was dispersed homogenously. A similar outcome was evaluated by Alam and coworkers where the NLCs particles were uniform and in spherical shape [45].

### 4.4. Powder X-Ray Diffraction (PXRD) Pattern Study

**Figure 11:**
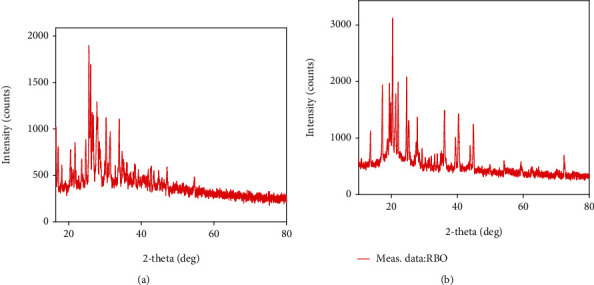
p-XRD of (a) pure RBO and (b) RBO-NLCs.

RBO was found to be crystalline in nature as it showed sharp, intense peaks located at 10.0, 14.8, 15.2, 15.7, 20.1, 22.6, and 23.4 ± 0.2°; 2 thetas in the powder X-ray diffraction spectrum are shown in Figure 11(a) [46]. The prepared RBO-NLCs were lyophilized to get the powder and used for p-XRD studies to see drug entrapment in the NLCs, as shown in Figure 11(b). The absence of the sharp and intense peaks of the drug in RBO-loaded NLCs indicates that the drug is completely entrapped within the NLCs or the unentrapped drug is converted into the amorphous state [47]. The mannitol used for lyophilization was converted into the amorphous state after freeze-drying and did not show any intense and sharp peaks [48, 49]. Yang and coworkers reported the development of gypenoside-loaded NLCs for oral delivery. The result found that typical peaks of the drug alone were missing in the XRD pattern of formed NLCs, in which attributed drugs in NLCs may appear in an amorphous state [50].

### 4.5. Structural Analysis by FT-IR Spectroscopy

**Figure 12:**
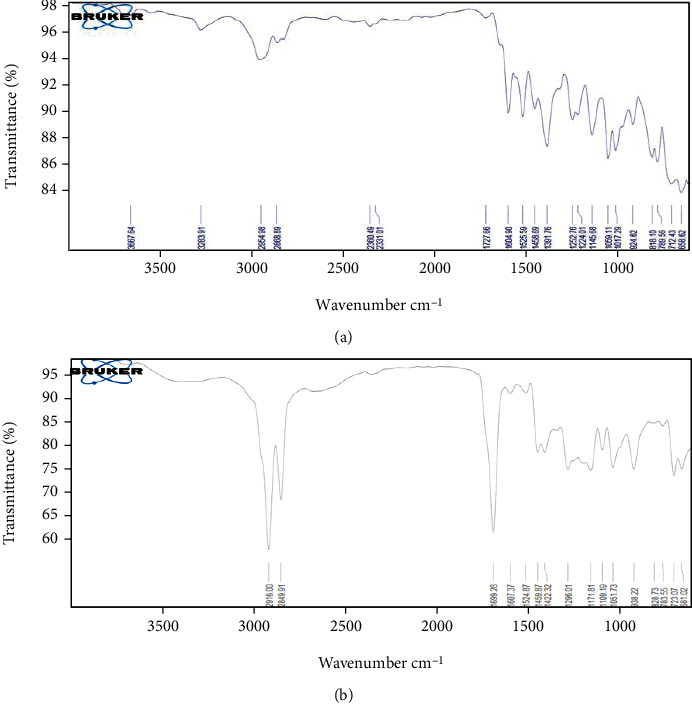
FT-IR spectra of (a) RBO (drug alone) and (b) RBO-NLCs.

The spectra obtained from FT-IR spectroscopy of drug alone and RBO-NLCs are demonstrated in Figures 12(a) and 12(b), respectively. The spectra of the drug alone have seen typical peaks at 3667.64 N–H stretch, 1727.66 C=O stretches, 1604.90–1458.69 C=C stretches, and 1391.78 C-N stretches. FTIR spectrum of RBO-NLCs showed a peak at 2916 C-H stretch, 2849.51 O-H stretch, and 1699.26 C=O stretch of carboxylic acid, which could be due to the existent of excipients in the NLCs formulation. In the FT-IR spectrum, the typical peak of RBO-NLCs was absent compared to the pure drug because of the complete entrapment of RBO inside the NLCs formulation [51]. Annu et al. have developed the chitosan nanoparticle. The result reported in the FT-IR study stated that the characteristic peaks of the drug in nanoparticles were absent compared to drug alone spectra, and it could be the possible reason for entrapment of the drug within the nanoformulation [52].

### 4.6. *In Vitro* Drug Release Study

**Figure 13:**
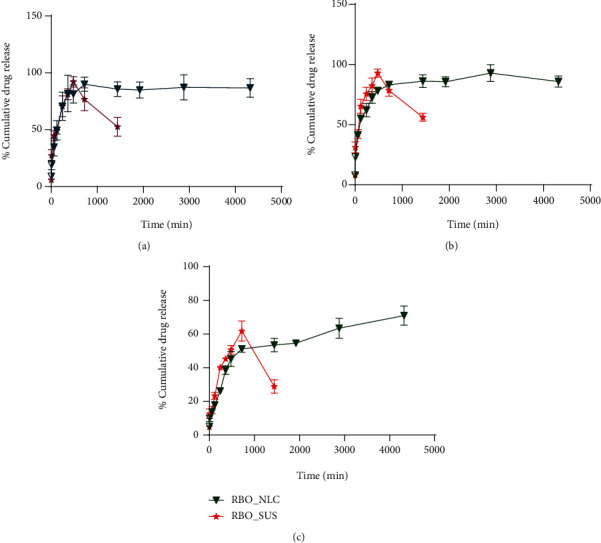
% CDR of RBO-NLCs and RBO-SUS in various buffers: (a) 0.1 N HCl buffer pH 1.2; (b) acetate buffer (AB) pH 4.5; (c) phosphate buffer saline (PBS) pH 6.8.

RBO-NLCs and RBO-SUS shown in Figure 13 indicated a sustained drug release at different pH 1.2, 4.5, and 6.8 from the initial time point as compared to RBO-SUS, which shows a gradual increase in release but not sustained for 24 h. The drug present inside the lipid matrix of NLCs undergoes surface erosion which is attributed to its sustained release from the formulation. Initial burst release in the NLCs was due to the unentrapped drug. The *t*_75%_ and *t*_50%_ were calculated for each drug formulation. The dissolution profile was compared in terms of % dissolution efficiency (%DE) and similarity factor (f2), as shown in Table 11. Results showed a higher %DE for NLCs formulation in pH 1.2, 4.5, and 6.8 compared with RBO-SUS.

Similarly, similarity factor, f2 was calculated. The f2 values below 50 for pH 1.2, 4.5, and 6.8 indicated the both RBO-NLCs and RBO-SUS release pattern were not similar. Sahibzada and coworkers investigated a similar comparison of drug nanoparticle dissolution parameters for the enchantment of oral bioavailability. The %DE and similarity factor f2 were calculated for two methods to prepare nanoparticles and compared for better dissolution [21].

### 4.7. Mechanism of Drug Release

The kinetic release model in the buffer of pH 1.2, 4.5, and 6.8 was calculated. Based on the *R*^2^ value, the appropriate kinetic model was chosen for the drug release mechanism from NLCs. So, the Korsmeyer-Peppas kinetic release model was selected as the *R*^2^ value was close to linearity compared to other kinetic release models. This model described release mechanisms from NLCs formulation, which described in three steps the diffusion of water, swelling, and dissolution of the matrix [53]. Duong and coworkers investigated the drug-loaded NLCs, and their drug release kinetic model was compared, and the mechanism was studied using the Korsmeyer-Peppas equation [18, 53, 54].

### 4.8. *In Vitro* Lipolysis Study

**Figure 14:**
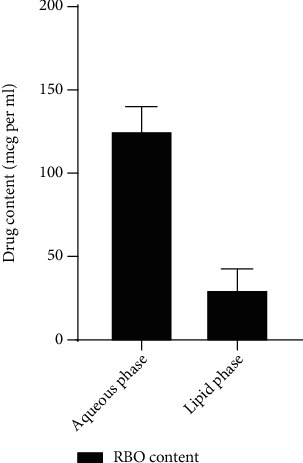
RBO content in different phases of lipolysis media.

The drug should be solubilized near the absorptive membrane so that it is available for systemic absorption. Therefore, the drug should solubilize in an aqueous layer in lipolysis media, and the same will not happen in the case of the sediment layer. The percentage of drug content in a different layer of aqueous and sediment is depicted in Figure 14. The high solubilization potential of the drug in the NLCs formulation in the aqueous layer indicated higher absorption, i.e., approximately 76% after orally administering the RBO-NLCs formulation. Furthermore, drug content in the sediment phase of RBO-NLCs was also calculated and found to be 18.47 ± 8.45%. This is because some of the solid lipids might not be digested, so the entrapped drug remains inside the solid lipid. Thus, the result established that RBO-NLCs enhanced its solubilization in the aqueous medium, which simulates the environment of GIT and the intestine. Hence, it can be used as *in vivo* fate of the RBO. A similar investigation was reported by Khan et al. and Rehman et al., in which the drug-loaded NLCs formulation found high drug content available for solubilization in the aqueous phase compared to the sediment phase [18, 55].

### 4.9. *In Vitro* Haemolysis Study

**Figure 15:**
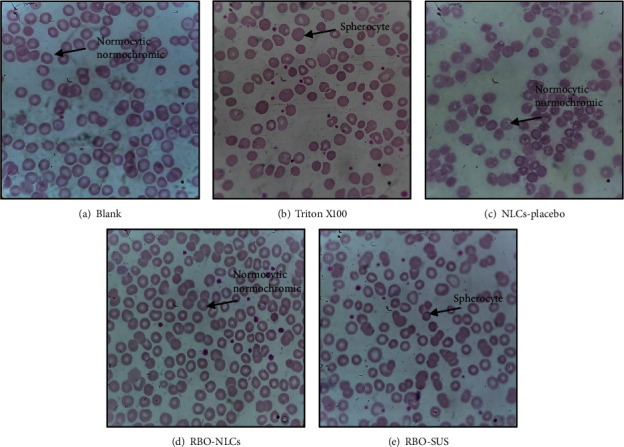
Haemolysis of red blood cells after being treated with RBO-NLCs formulations and RBO-SUS.

Haemolysis is detected in red blood cells treated with experimental agents, as shown in Figure 15. The positive control sample showed the least variability in red blood cells with respect to their structure or shape, which was almost similar to the sample treated with NLCs-placebo as the haemolysis count was significantly high in the case of Triton X100 treated sample which destabilizes erythrocytes membrane. The RBO-NLCs formulation showed well intact red blood cells, which were again similar to the NLCs placebo-treated sample (% haemolysis of 1.855%). However, slight structure modification occurred in the case of the RBO-SUS sample (% haemolysis of 2.712%) treated red blood cells, which disrupts the membrane of red blood cells. The result concluded that the NLCs formulation of RBO was safer compared to a pure drug suspension. So, the result comprises the *in vivo* fate of NLCs formulation of the selected drug. A similar investigation was reported by Chauhan and associates in which the curcumin nanoformulation evaluated their anticancer potential and evaluated direct nanoformulation erythrocyte interaction in which membrane disruption directly measures the toxicity of nanoformulation [56].

### 4.10. *In Vitro* Gut Permeation Study

**Figure 16:**
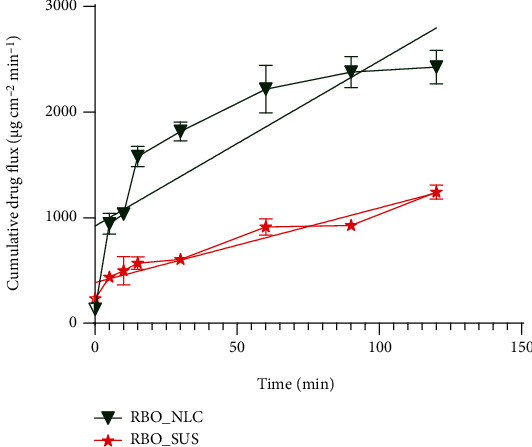
The flux of drug permeated in rat intestine at different time point intervals.

The intestinal permeability of RBO-NLCs and RBO-SUS is shown in Figure 16. After 2 h study setup, the flux of the drug in the rat intestine was calculated. The flux for RBO-NLCs and RBO-SUS formulations was found to be 2426.98 ± 157.77 and 1241.77 ± 68.70 *μ*g/cm^2^, respectively. The apparent permeability coefficient was found to be 75.86 ± 2.46 and 38.81 ± 1.07 cm min^−1^ for RBO-NLCs and RBO-SUS, respectively. The smaller particle size and permeation enhancer (lipids) of RBO-NLCs enhanced the drug's permeability compared to the RBO-SUS. The reason can be owed to the P-gp inhibitory activity of Solutol HS 15, which inhibits the drug efflux, thereby enhancing the permeability. Almost two times of drug release were observed in RBO-NLCs in contrast to the RBO-SUS formulation [12]. Wu and coworkers investigated the repaglinide-loaded NLCs formulation which demonstrated the membrane permeability compared with their solution. Drug-loaded NLCs with small particle size easily pass the mucus layer and intestinal epithelial cell layer compared with large NLCs [57].

### 4.11. Depth Permeation Study by Confocal Microscopy

**Figure 17:**
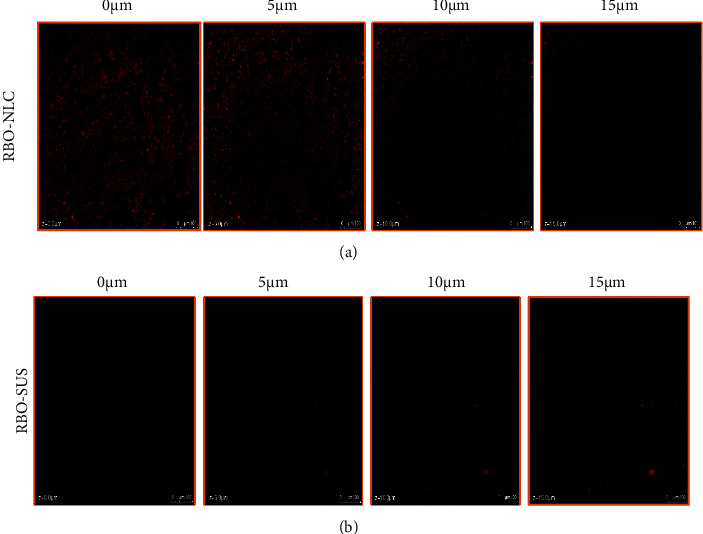
Depth of penetration shown in small intestine cells by using CLSM imaging after 2 hours of administration of (a) RBO-NLCs and (b) RBO-SUS.

The confocal microscopy images of rat intestine with Rhodamine B dye, as shown in Figure 17, showed the depth of penetration of RBO-NLCs and RBO-SUS, respectively, after 2 h of dose administration. The depth of penetration measured through the *z*-axis is 15 *μ*m for both formulations. The data obtained distinctly indicated the intensified fluorescence in RBO-NLCs in contrast to RBO-SUS even after 15 *μ*m of depth. The result signifies greater penetration of RBO-NLCs, which could be the possible reason for the small particle size of NLCs and the role of lipids and surfactants as penetration enhancers [58]. Soni and coworkers reported the sulforaphane-loaded NLCs, and the result suggested that the depth of permeation of the drug was measured in the intestinal lumen by the depth of fluorescence permeated. Drug-loaded NLCs penetrated more as compared to drug solutions [58].

### 4.12. Cell Line Study

#### 4.12.1. Effect of RBO on MCF-7 Breast Cancer Cells

**Figure 18:**
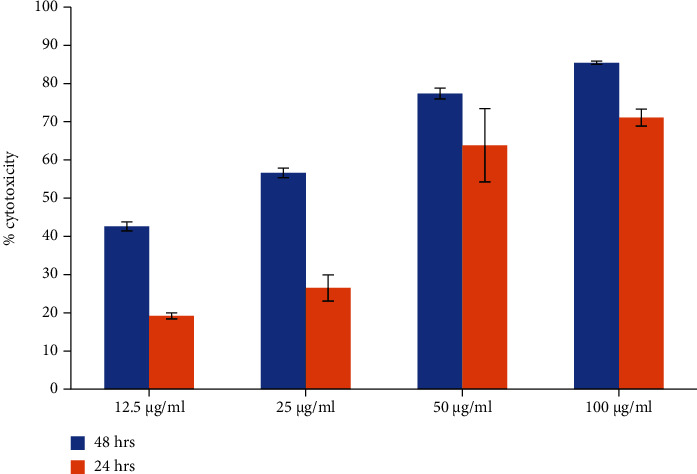
Dose-dependent cytotoxicity assay of RBO on MCF-7 cell line at 24 hrs and 48 hrs by MTT.

**Figure 19:**
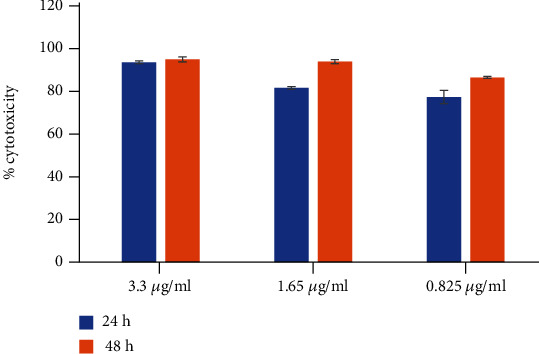
Dose-dependent cytotoxicity assay of RBO-NLCs on MCF-7 cell line at 24 hrs and 48 hrs by MTT.

The antitumor activity of RBO against the MCF-7 cells was investigated by MTT assay. As shown in Figure 18, RBO exhibited a broad spectrum of inhibition against cancer cell lines in a dose-dependent manner. The result from the MTT assay, the IC_50_ value (*μ*g/ml) of RBO post-24 hrs and 48 hrs of treatment against the MCF-7 cell line at 37°C at 5% CO_2_, was found to be 39.51 and 13.36 *μ*g/ml, respectively. After calculation of the IC_50_ value, similarly dose-dependent toxicity of the RBO-NLCs formulation was done as shown in Figure 19. The % cytotoxicity was significantly enhanced in the case of nanoformulation with lower IC_50_ value of 0.531 and 0.474 *μ*g/ml post-24 hrs and 48 hrs, respectively. So, by formulating into NLCs of the drug, the cytotoxicity was enhanced and reduced their IC_50_ value by 74 times and 28 times after 24 h and 48 h, respectively. A similar study was also conducted by Marinelli and coworkers in which RBO alone or in combination with Everolimus was investigated in breast cancer lines [59].

### 4.13. *In Vivo* Pharmacokinetic Study

**Figure 20:**
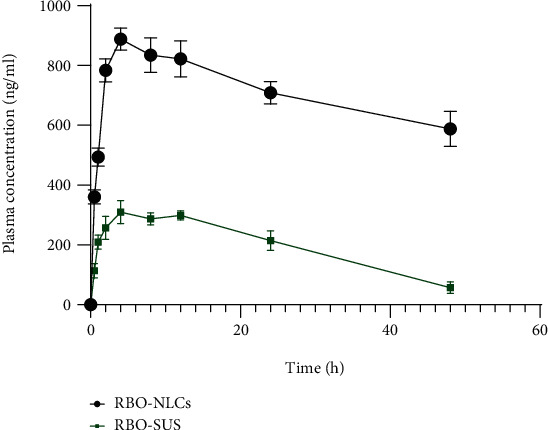
The plasma concentration versus time profile curve of RBO-SUS and RBO-NLCs after oral administration. Data expressed in mean ± standard deviation (*n* = 3).

*In vivo* pharmacokinetic study was conducted in female Wistar rats for the determination of bioavailability data, as depicted into Figure 20 after the oral administration of RBO suspension and RBO-loaded NLCs. The peak plasma concentration (*C*_max_) and time of occurrence of the same (*T*_max_) were calculated directly from the drug plasma concentration versus time profile graph. The significant parameters are provided in Table 12 calculated by the linear trapezoidal method using the PK solver add-in tools in Microsoft excel. RBO belongs to the BCS IV drug category which makes it essential to develop a novel drug delivery system so that NLCs can overcome solubility as well the permeability issue. The comparative data of RBO suspension and RBO-NLCs are summarized in Table 12 where *C*_max_ and *T*_max_ of RBO suspension and RBO-NLCs were found to be 311.90 ± 36.70 ng/ml and 4.00 h and 897.92 ± 28.14 ng/ml and 4.00 h, respectively. The higher *C*_max_ of RBO-NLCs indicated the higher absorption due to their nanosize and lipophilic characteristic of NLCs which is also responsible for micellar solubilization which enhances the lymphatic uptake through Peyer's patches present in the small intestine [60, 61]. The higher *T*_max_ also suggested the prolonged and sustained drug release. The *in vivo* result corroborated with *in vitro* drug release of RBO in terms of sustained and longer period of drug release. Higher absorption is achieved due to more of the drug being entrapped inside the lipidic nanoparticles, and again, the micellar solubilization leads to conversion of nanoparticles into chylomicrons which enhance the lymphatic uptake and hence bypass the hepatic first pass metabolism. From the calculated data, a 3.54-fold increase in the bioavailability of RBO-NLCs was observed as compared to the RBO suspension. Data showed statistical difference at ^∗^*p* < 0.001 between RBO-SUS and RBO-NLCs.

### 4.14. Stability Study

**Figure 21:**
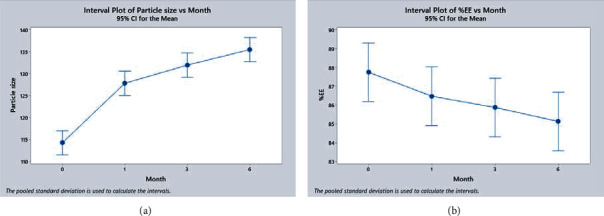
Change in (a) particle size and (b) % entrapment efficiency upon stability study for 6 months.

The parameters of optimized NLCs on the stability study were evaluated as per the ICH guidelines, and change in any physical and chemical was evaluated as shown in Table 13. A freshly prepared sample was kept for stability study after determining their particle size, and PDI, which was 114.23 ± 2.75 nm and 0.649 ± 0.043, respectively, and % entrapment efficiency was 87.75 ± 1.79% on 0 days. As the time point from 1 month to 6 months of stability, the particle size increases from 114.23 to 135.44 nm, as shown in Figure 21(a). It might be because of the swelling of NLCs nanoparticles upon a long period of storage. The entrapment efficiency decreases during the storage time, as shown in Figure 21(b). It might be because solid and liquid lipid integrity decreases and swelling of nanoparticles causes the leakage of the drug, reflecting the decrease in entrapment efficiency. Thus, the drug-loaded NLCs showed good physical and chemical stability upon long storage [45]. The changes in particle size and entrapment efficiency are shown in Figures 21(a) and 21(b), respectively, have drawn by using software Minitab 19, Philadelphia, USA. All data obtained from stability corresponded to the preceding research done by Nabi and associates in which they have formulated NLCs using the BBD and stability study done for 90 days [29]

## 5. Conclusion

RBO loaded NLCs was prepared using solvent evaporation followed by the probe sonication method. The NLCs formulation was optimized using the Box-Behnken design response surface method. The prepared NLCs exhibited mean particle size of 114.23 ± 2.75 nm, mean polydispersity index of 0.649 ± 0.043, and high entrapment efficiency of 87.7 ± 1.79%. TEM images also suggested uniform size and distribution. The *in vitro* drug release from the NLCs nanoformulation shows a sustained release for a long time (up to 72 h), unlike drug suspension, which is only up to 24 h of drug release, and it could not maintain the sustained drug release for a longer period. The *in vitro* lipolysis study indicated the drug availability after released from lipid-encapsulated drug to the absorption site. The *in vitro* haemolysis study suggested that the NLCs formulation of the given drug was safe compared to the pure drug. The *in vitro* permeation study showed an increase in depth of the NLCs formulation due to the lipid used in the formulation, which enhances the permeation as compared to a drug suspension. The cell lines in the MCF-7 study which significantly reduced the dose in the case of the NLCs formulation required to produce similar cytotoxicity when pure drug was used. The findings of the pharmacokinetics study by the *in vivo* study in female Wistar rats of the RBO-NLCs were carried out which enhance its bioavailability by 3.54 times as compared to the RBO-SUS.

## Figures and Tables

**Table 1:** Release kinetic equation for the analysis mechanism of drug release.

Kinetic model	Equation
Hixson-Crowell	3√*Q*_*o*_ − 3√*Q*_*t*_ = *Kt*
1st order	ln*Q*_*t*_ = ln*Q*_*o*_ + *Kt*
Higuchi	$Q_{o}-Q_{t}={Kt}^{\frac{1}{2}}$
Korsmeyer-Peppas	Log(*Q*_*o*_ − *Q*_*t*_) = log*K* + *n*log*t*

*Q*_*o*_ is the amount of drug present initially, *Q*_*t*_ is the drug left over after time *t*, and *K* is released constantly.

**Table 2:** List of solid and liquid lipids and their solubility parameters to dissolve RBO.

Liquid lipids	Solubility (mg mL^−1^)		Solid lipids	Solubility (mg g^−1^)	
	Mean	SD		Mean	SD
Labrafil M 1944 CS	4.6856	0.0605	Campritol 888 ATO	14.00	2.022
Labrafil M 2125 CS	10.3348	0.3013	Precirol ATO 5	13.87	0.351
Lauroglycol FCC	2.1232	0.0419	Stearic acid	95.10	5.384
Capmul MCM- C8	8.1974	0.1463	Gelot 64	14.70	2.358
Capmul MCM- L8	2.0013	0.1012	Capmul MCM C10	77.33	2.281
Capmul PG-12	3.3194	0.1596	Gelucire 44/14	20.90	2.402
Caprol PGE-860	3.3646	0.0818	Gelucire 44/01	20.40	2.381
Maisine 351	8.9596	0.1655			
Peceol	3.6687	0.0766
Plurol oleique	11.0081	0.2399
Plurol oleique CC 497	3.3744	0.1525
Sesame oil	4.1888	0.1101

**Table 3:** Results of miscibility study between liquid lipids and solid lipids.

Solid lipid	Liquid lipid	Observation	Inferences
Stearic acid	Labrafil M 2125 CS	After 48 h, separate layers were observed.	Failed
	Capmul MCM-C8	Uniform distribution and no phase separation were observed.	Good compatibility
	Maisine 351	Turbidity and phase separation was found after 48 h.	Failed
	Plurol oleique		
Capmul MCM C10	Labrafil M 2125 CS	No phase separation was observed.	Passed
	Capmul MCM-C8		
	Maisine 351	After 48 h, all remain in the liquid phase.	Failed
	Plurol oleique		

**Table 4:** Observational study for the selection of surfactants.

Name of surfactants	% transmittance
Cremophor EL	64.23
Cremophor RH 40	91.61
Poloxamer 188	92.04
Solutol HS 15	98.28
Tween 20	45.95
Tween 80	63.69

**Table 5:** Quality target product profile of the nanostructured lipidic carrier.

QTPP parameters	Target to achieve	Justification
Dosage type	Nanoformulation	Escalate the drug permeation/bioavailability
Route of administration	Oral	Easy to use/lymphatic uptake/stability/minimize side effects
Physical state	Lyophilized powder	Easy administration/physical aspect
Physiochemical characterization	Entrapment efficiency	Drug loading assurance
	Particle size	Influence the permeation and absorption
	Zeta potential	Product stability assurance
Pharmacokinetics	Absorption	Required to achieve the desired efficacy
	Distribution	
	Metabolism and targeting	

**Table 6:** List of various CQAs affecting the therapeutic efficacy of RBO-NLCs.

CQA parameters	Target to achieve	Justification
Particle size	100-200 nm	Assure the absorption, hence the increase in bioavailability
Entrapment efficiency	≥70%	To reach the optimum therapeutic efficacy
PDI	≤0.7	Uniform drug distribution, hence content uniformity

**Table 7:** Different variables selected for Box-Behnken design.

Independent variables	Factors	Unit	Level	
			Low	High
A1	Binary mixture	% *w*/*v*	1	3
A2	Surfactant	% *w*/*v*	1	3
A3	Sonication	Min	1	5

**Table 8:** Experimental runs and observed responses from BBD.

Std	Runs	Factor 1	Factor 2	Factor 3	Response 1	Response 2	Response 3
		Binary mixture (% *w*/*v*)	Surfactant (% *w*/*v*)	Sonication time (min)	Particle size (nm)	PDI	Entrapment efficiency (% *w*/*w*)
14	1	3	1	3	656	0.653	94.5
13	2	2	2	3	211.9	0.562	89.9
17	3	2	3	5	179.87	0.554	89.99
16	4	1	2	1	230.7	0.497	87.1
1	5	1	1	3	212.54	0.499	85.9
9	6	1	2	5	152.32	0.618	86.1
3	7	2	3	1	221.92	0.5	87.76
15	8	3	3	3	276.53	0.621	96.12
8	9	2	2	3	225.9	0.549	87.98
11	10	2	2	3	234.8	0.541	89.42
7	11	2	1	5	465.71	0.487	87.91
12	12	2	2	3	212.7	0.502	88.4
6	13	1	3	3	114.23	0.649	87.7
4	14	3	2	5	543.73	0.597	95.4
5	15	3	2	1	419.34	0.682	96.71
2	16	2	2	3	211.9	0.562	89.9
10	17	2	1	1	396.4	0.512	90.14

**Table 9:** Interpretation of regression value of dependent variable responses B1, B2, and B3.

Dependent variables	*R* ^2^	*R* ^2^		Adequate precision	SD	% CV
		Adjusted value	Predicted value			
Particle size (B1)	0.9983	0.9962	0.9908	74.6866	9.27	3.17
PDI (B2)	0.9500	0.8856	0.7640	12.2250	0.0211	3.75
EE (B3)	0.9692	0.9297	0.7403	14.2001	0.9234	1.03

**Table 10:** Point prediction method to attain predicted and actual response (maximum entrapment efficiency, optimum PDI, and minimum particle size) [12].

	Lipid (% *w*/*v*)	Surfactant (% *w*/*v*)	Sonication time (min)	Particle size (nm)	Entrapment efficiency (%)	PDI
Predicted	1.0	3.0	3.0	219.44	89.12	0.543
Actual				117.00	87.70	0.600

**Table 11:** Dissolution profile comparison for NLCs and suspension formulation in different dissolution media.

Parameters	Formulation	0.1 N HCL (pH 1.2)	AB (pH 4.5)	PBS (pH 6.8)
%DE	RBO-NLCs	83.98 ± 7.69	84.37 ± 2.97	55.98 ± 3.16
	RBO-SUS	68.30 ± 4.64	71.33 ± 3.06	43.55 ± 1.48
f2 similarity factor	—	43.89	43.76	48.52
*T*_50%_ (h)	RBO-NLCs	2.03	1.38	11.27
	RBO-SUS	2.05	1.20	—
*T*_75%_ (h)	RBO-NLCs	4.47	6.53	—
	RBO-SUS	4.49	3.53	—

**Table 12:** Pharmacokinetic parameters of RBO-SUS and RBO-NLCs.

Pharmacokinetic parameters	RBO-SUS	RBO-NLCs
*C*_max_ (ng/mL)	311.90 ± 36.70^∗^	897.92 ± 28.14^∗^
*T*_max_ (h)	4.00	4.00
AUC_0−*t*_ (ng h/mL)	9620.75 ± 781.21^∗^	34117.67 ± 548.20^∗^
AUC_0−*α*_ (ng h/mL)	10924.97 ± 751.39^∗^	94903.96 ± 21646.64^∗^
*t*_1/2_ (h)	14.95 ± 3.85	70.53 ± 18.82

Data represented as mean ± standard deviation (*n* = 3) with statistical difference at ^∗^p < 0.001.

**Table 13:** Result of stability study for RBO-NLCs at 25 ± 2°C/60 ± 5% RH (*n* = 3).

Time (month)	Phase separation	Sedimentation	Particle size		PDI		Entrapment efficiency	
0	No	No	nm	SD	PDI	SD	%EE	SD
			114.23	2.75	0.649	0.043	87.75	1.79
1	No	No	127.78	1.26	0.711	0.067	86.47	0.91
3	No	No	131.91	2.50	0.703	0.054	85.87	0.79
6	No	No	135.44	1.31	0.707	0.067	85.13	0.91

## Data Availability

All data are provided in full in the Results and Discussion section of this paper.

## Abbreviations

RBO: Ribociclib
NLCs: Nanostructured lipid carriers
P-gp: P-Glycoprotein
BBD: Box-Behnken design
PDI: Polydispersity index
EE: Entrapment efficiency
TEM: Transmission electron microscopy
*R* ^2^: Regression value
CDK: Cyclin-dependent kinase
IUPAC: International Union of Pure and Applied Chemistry
HLB: Hydrophilic and lipophilic balance
rcf: Relative centrifugal force
UV: Ultraviolet
QbD: Quality by design
p-XRD: Powder X-ray diffraction
FT-IR: Fourier-transform infrared spectroscopy.
